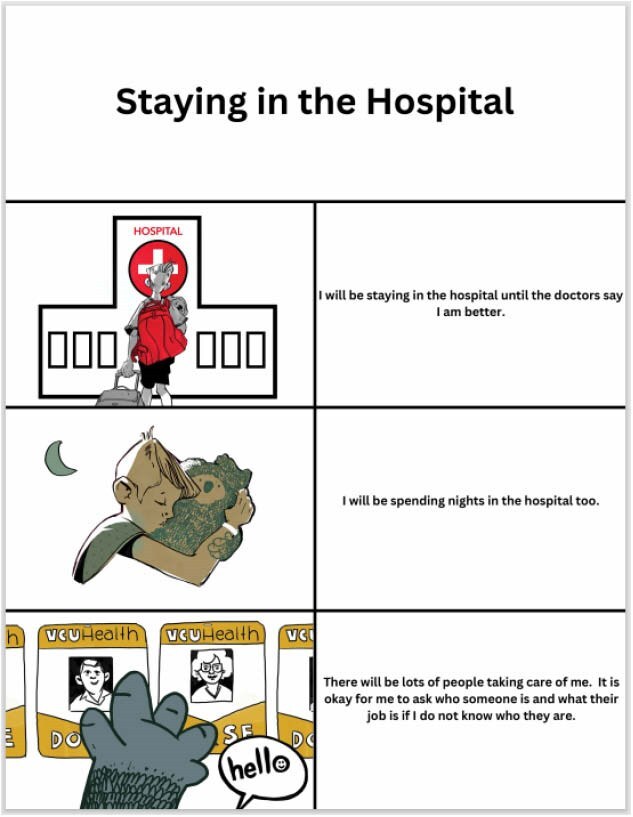# 984 Social Stories on the Burn Unit: Improving Communication with Neurodiverse Patients

**DOI:** 10.1093/jbcr/iraf019.515

**Published:** 2025-04-01

**Authors:** Elizabeth Bowers, Arleta Brehm, Michael Feldman, Tiffany Lord, Sterling Hundley

**Affiliations:** Evans-Haynes Burn Center at VCU Health; Virginia Commonwealth University; Virginia Commonwealth University; Virginia Commonwealth University; Virginia Commonwealth University

## Abstract

**Introduction:**

Medical personnel frequently report that they possess limited training, skills, and resources to effectively interact with neurodiverse patients. Neurodiverse individuals report poorer quality of healthcare and worse overall health than neurotypical counterparts. One method for improved communication with neurodiverse patients is social stories. They introduce readers to unfamiliar situations utilizing clear, brief language accompanied by a simple picture or symbol. They provide the reader with a linear set of events, expected behaviors, and outcomes.

Our burn center is a 16-bed unit that serves adult and pediatric patients, with an average of 550 admissions a year. Several cases have made it clear that we need an improved approach to communication with the neurodiverse population.

**Methods:**

Interviews were conducted with burn center staff regarding their comfort level in communicating and interacting with neurodiverse patients. Additional interviews were held with subject matter experts regarding effective communication methods in current practice. Lastly, a literature review was performed using PubMed.

**Results:**

Staff interviews confirmed a perceived lack of education, knowledge, and resources concerning neurodiverse patients. They also reinforced the desire for better communication tools. Literature and subject matter expert input highlighted the use of social stories to promote desirable behaviors in the medical setting. We created 11 social stories through a collaboration with a Professor of Arts at the University. They include: “what are burns?, wound care, intravenous catheter placement, lab draws, nasogastric tube placement, physical and occupational therapy, tomorrow I have surgery, urinary (pee) catheter, changing rooms: moving to the burn unit, coping strategies, and staying in the hospital.”

**Conclusions:**

Neurodiverse patients are at risk for poorer health care outcomes versus their neurotypical counterparts. They are also at risk for burn injuries. This, in addition to a lack of training in how to communicate with the population, is an opportunity for our Burn Center. We have addressed this critical issue with Social Stories, which we believe are a resource for all of those who manage neurodiverse patients.

**Applicability of Research to Practice:**

Social stories are a needed communication tool that provides medical personnel with a way to inform neurodiverse individuals regarding their care. This QI project demonstrates that burn centers can successfully develop burn-specific social stories for use with neurodiverse patients.

**Funding for the Study:**

A hospital auxiliary grant was applied for and was partially awarded. We are currently investigating the establishment of an art class through the university designed to complete the remainder of the social stories.